# Epigenetic insights into physiological resilience: Multigenerational readouts of CO_2_-induced seawater acidification effects on fish embryos

**DOI:** 10.1016/j.isci.2025.113187

**Published:** 2025-07-26

**Authors:** Tzu-Yen Liu, Jia-Jiun Yan, Ying-Jey Guh, Oki Hayasaka, Li-Yih Lin, Pung-Pung Hwang, Guan-Chung Wu, Ming-Tsung Chung, Yung-Che Tseng

**Affiliations:** 1 Institute of Oceanography, National Taiwan University, Taipei City, Taiwan; 2 Institute of Cellular and Organismic Biology, Academia Sinica, Taipei City, Taiwan; 3 Faculty of Fisheries, Kagoshima University, Kagoshima, Japan; 4 Department of Life Science, School of Life Sciences, National Taiwan Normal University, Taipei City, Taiwan; 5 Department of Aquaculture, National Taiwan Ocean University, Keelung, Taiwan; 6 Center of Excellence for the Oceans, National Taiwan Ocean University, Keelung, Taiwan; 7 Faculty of Laboratory of Physiology, Atmosphere and Ocean Research Institute, The University of Tokyo, Chiba, Japan

**Keywords:** Aquatic biology, Aquatic science, Ichthyology, Marine geochemistry, Water geochemistry

## Abstract

Anthropogenic CO_2_ emissions are acidifying oceans, threatening marine organisms during early development. We investigated multigenerational effects of projected 2100 acidification (pH 7.6) on marine medaka (*Oryzias melastigma*) embryos across three generations using integrated phenotypic, physiological, transcriptomic, and epigenetic analyses. Prolonged acidification altered developmental trajectories, with F2 embryos showing size reductions. Metabolic responses were generation-specific: F0 embryos displayed decreased ammonium excretion, while F1 and F2 maintained stable profiles. Transcriptomic analysis revealed generational changes in neurotransmission, ion regulation, and epigenetic pathways. F2 embryos exhibited attenuated transcriptional perturbations and partial restoration of acid-base homeostasis, suggesting enhanced adaptability. Adaptive gene expression correlated with hypomethylation recovery of ion transport genes AE1a and NHE2 in F2 embryos. Increased hypomethylated AE1a promoter CpG sites in F1 and F2 generations aligned with elevated transcription, indicating epigenetically-driven enhancement. These results demonstrate epigenetic control’s crucial role in multigenerational plasticity and adaptive responses to ocean acidification.

## Highlights

• Ocean acidification causes generation-specific developmental and metabolic changes
• F2 embryos show enhanced resilience through transcriptional recovery mechanisms
• Hypomethylation of ion transport genes drives adaptive acid-base regulation
• Epigenetic inheritance facilitates multigenerational acclimation to acidification

## Graphical abstract

**Figure:**
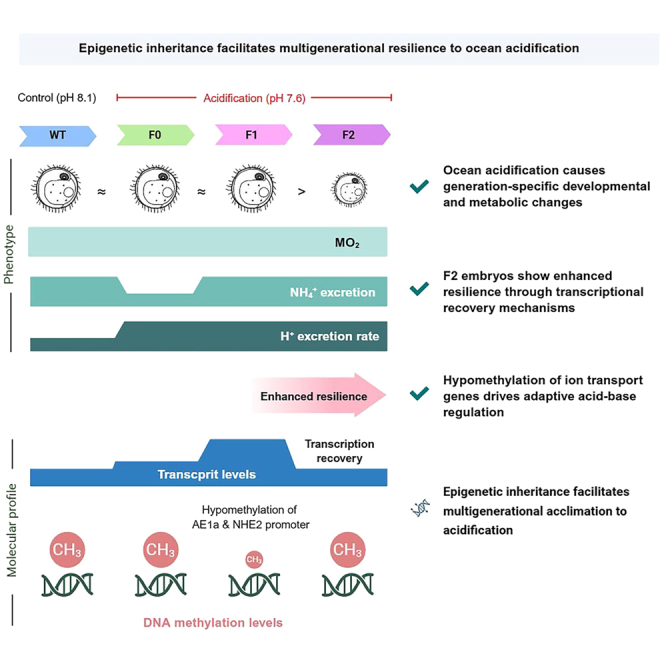

## Introduction

Anthropogenic CO_2_ emissions, predominantly from fossil fuels,^1^ represent a significant threat to marine conditions, particularly through the possibility of a decrease in pH levels. Since the Industrial Revolution,^2^^,^^3^ the oceans have absorbed considerable CO_2_ emissions. Present projections indicate that by the end of this century, the oceans may have absorbed the total amount of CO_2_ emitted since the beginning of the industrial era. Under the RCP8.5 scenario, which assumes greenhouse gas emissions will continue to rise unchecked, the pH of the oceans is expected to drop by as much as 0.43 units by the end of the 21^st^ century.^4^

The ocean’s marine biosphere is nearing historically high acidity levels, mainly due to atmospheric CO_2_ levels surpassing their natural range.^5^ Ocean acidification (OA) is a particularly significant and wide-ranging effect of CO_2_, which has a long atmospheric lifetime and persists in the atmosphere for centuries.^6^ Research has shown that marine organisms can adapt to OA through physiological and biochemical mechanisms.^7^^,^^8^ Additionally, the long-term impacts of continuous CO_2_ presence have frequently been overlooked because previous research primarily focused on short-term or single-generation exposure to these changes.^9^^,^^10^ However, a growing body of evidence emphasizes the creation and acceleration of transgenerational ocean acidification (TGA),^11^^,^^12^ which describes the effects of rising CO_2_ levels on multiple marine generations. Recent scientific efforts have identified potential adaptive mechanisms, such as intra-, *trans*-, or multi-generational plasticity, aimed at counteracting acidification.^13^^,^^14^ These mechanisms could have involved the transfer of nutrients, hormones, or epigenetic markers that affect gene regulation across multiple generations. Observations of interest include the adaptive responses of the offspring of marine organisms, such as the cinnamon clownfish (*Amphiprion melanopus*) and the Sydney rock oysters (*Saccostrea glomerata*), which occur when parents are conditioned in an acidic environment.^15^^,^^16^ These observations highlighted the importance of multigenerational plasticity.^17^ Studies conducted outside laboratory confines on species, like the Atlantic silversides (*Menidia menidia*), have supported these findings.^18^ The adaptive features, referred to as *trans*- or multi-generational plasticity, indicate that the adaptability of offspring can be enhanced without alterations in DNA sequences in situations where parents face fluctuating environments.^19^^,^^20^ While precise molecular processes that drive this adaptability are still being carefully investigated, teleosts serve as an ideal model system for the inquiry of such adaptive responses. Teleosts, essential components of marine ecosystems and contributions to human food security, show phenomenal physiological adaptability.^21^^,^^22^ The early life stages of these organisms are particularly illuminating for the study of environmental adaptation mechanisms,^23^^,^^24^ as this period is a critical developmental window during which fundamental physiological systems, such as acid-base regulation and osmoregulation, are being established. Ionocytes, the primary cellular mediators of acid-base homeostasis, encounter critical functional maturation on the yolk sac epithelium and body surface during early development.^25^^,^^26^^,^^27^ This presents a perfect opportunity to investigate the role of environmental acidification in the establishment of essential physiological processes.

**Figure 1:**
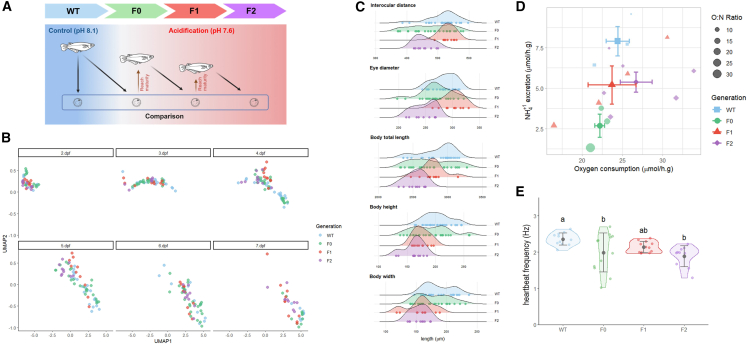
Experimental design and phenological patterns of marine medaka (*Oryzias melastigma*) embryos under control and elevated CO_2_-induced acidification across multiple generations (A) A schematic representation of the multigenerational experimental design. The control group, referred to as the wild-type (WT), was maintained in normal seawater with a pH of 8.1. In contrast, three consecutive generations, namely F0, F1, and F2, were subjected to conditions of acidified seawater with a pH of 7.6, beginning from the fertilized egg stage and continuing through to sexual maturity. The unidirectional comparison arrow delineates the common reference-point design, whereby all generations exposed to acidification were systematically evaluated against the established baseline of the control wild-type. (B) UMAP analysis of embryonic phenological patterns from 2 to 7 days post-fertilization (dpf) in control (WT, blue) and acidified groups (F0, green; F1, red; F2, purple). (C) Morphometric parameters of 5 dpf embryos, including interocular distance, eye diameter, total body length, body height, and body width, in control (WT) and acidification groups (F0, F1, and F2). (D) Metabolic profiles of 5 dpf embryos, characterized by oxygen consumption (μmol/mg), NH_4_^+^ excretion (μmol/mg), and O:N ratio. (E) Heartbeat frequency (*n* = 10–12 per group) of 5 dpf embryos in control and acidified groups. Data are shown as mean ± SD. Statistical analysis was performed in a stepwise manner where normality was tested using the Shapiro-Wilk test, and the homogeneity of variance was examined through Levene’s test. For normally distributed data with homogenous variances, use a one-way ANOVA with Tukey’s post-hoc test. For heterogeneous variances, use Welch’s ANOVA with the Games-Howell post-hoc test. For non-normally distributed data, the Kruskal-Wallis test was used with the appropriate post-hoc comparisons. Different letters indicate significant differences between groups (*p* < 0.05). Statistical details are provided in Tables S1, S2, and S3.

Maintaining ionic and acid-base homeostasis is physiologically essential for marine organisms. Ocean acidification poses a significant threat to marine life, particularly fish and corals, by disrupting their ionic equilibrium and physiological processes.^24^^,^^28^^,^^29^ These effects can include reduced metabolic rates, impaired neurosensory function, decreased larval settlement and post-settlement growth, and compromised immune system function.^24^^,^^29^^,^^30^^,^^31^ In the commercial fish *Argyrosomus regius*, studies have documented physiological acclimation to ocean acidification, though such responses typically involve complex energetic trade-offs, and their long-term implications under multiple environmental stressors remain to be fully understood.^32^ Indeed, teleosts have developed complex processes that allow epithelial cells to secrete more protons (H^+^) and store more bicarbonate (HCO_3_^−^) in response to acidifying environments.^33^^,^^34^ From a broader perspective, the ionic shifts, acid-base regulation, and carbon capture facilitated by fish play a crucial role in maintaining the overall health and sustainability of marine ecosystems.^35^ As fish serve as a vital protein source for humans and an essential component of marine ecology,^36^ understanding these acclimation mechanisms is of utmost importance. Despite the potential for acclimation responses, the long-term consequences of ocean acidification on marine life remain uncertain, emphasizing the need for continued research and conservation efforts. To identify the consequences of environmental acidification on the early life stages of a representative marine fish model,^37^ this study employs a genetic model species, marine medaka (*Oryzias melastigma*). By understanding the potential epigenetic inheritance of physiological responses in CO_2_-acidified acclimated offspring during their early life stages (Figure 1A), our findings are anticipated to illuminate the process for effective mitigation strategies against environmental acidification.

## Results

### Acidification alters key growth trajectories during development

To comprehensively evaluate the impacts of acidification on developmental trajectories, we conducted a multifaceted assessment of key growth parameters representing biometric characteristics throughout ontogenetic maturation in the marine medaka. Uniform Manifold Approximation and Projection (UMAP) analysis integrated developmental measurements across generations, enabling statistically distinct comparisons between generational phenotypes. Among developmental stages, 5 dpf embryos displayed the earliest disparity of embryonic phenological patterns between generations (Figures 1B and S1A). Given 5 dpf embryos showed the earliest acidification-induced divergence, subsequent examinations of ontogeny monitoring, metabolic and molecular responses focused predominantly on this stage.

Examinations of ocular morphologies and body trunks in 5 dpf embryos revealed generation-specific responses to acidification when compared to their contemporary control groups (Figure 1C; Table S1). While F0 embryos showed no significant difference in interocular distance compared to wild-types (*p* = 0.105), F2 embryos exhibited significantly reduced ocular measurements (*p* < 0.001 compared to WT; *p* < 0.001 compared to F1). Principal-component analysis (PCA) further resolved generational distinctions by consolidating developmental parameters into composite components (Figure S1B). The three primary principal components (PCs) collectively accounted for 90% of the total variance in 5 dpf embryos. PC1 explained 54% of the variance and represented the overall reduced phenotype of F2 embryos across growth parameters. PC2 accounted for 25% of the variance and reflected changes in ocular distance and body height. PC3 explained 11% of the variance, primarily reflecting ocular morphologies and body trunk.

### Acidification perturbs physiological homeostasis in early embryonic development

Oxygen consumption and ammonium excretion rates were analyzed in 5 dpf embryos (Figure 1D). One-way ANOVA indicated no significant differences in oxygen consumption across groups (*F*(3, 12) = 0.805, *p* = 0.5149, Table S2). Analysis of NH_4_^+^ excretion revealed a significant reduction in the F0 generation compared to wild-type (*p* < 0.05), whereas the F1 and F2 groups showed intermediate levels that were not significantly different from either wild-type or F0. This generation-specific change in NH_4_^+^ excretion indicated an initial adaptation in nitrogen metabolism caused by exposure to acidification. Besides, heartbeat assessments revealed pronounced impacts on pulse frequency (Figure 1E; Table S3). Prolonged high CO_2_ exposure elicited generational differences in cardiac function, decreasing heart rate in F2 embryos while elevating heart pulse data irregularity in the F0 group (Figure 1E). Together, these findings demonstrated that multigenerational exposure to acidified conditions alters basal energy metabolism and cardiac function in early embryonic stages.

### Acidification reshapes the transcriptomic landscapes in developing embryos

**Figure 2:**
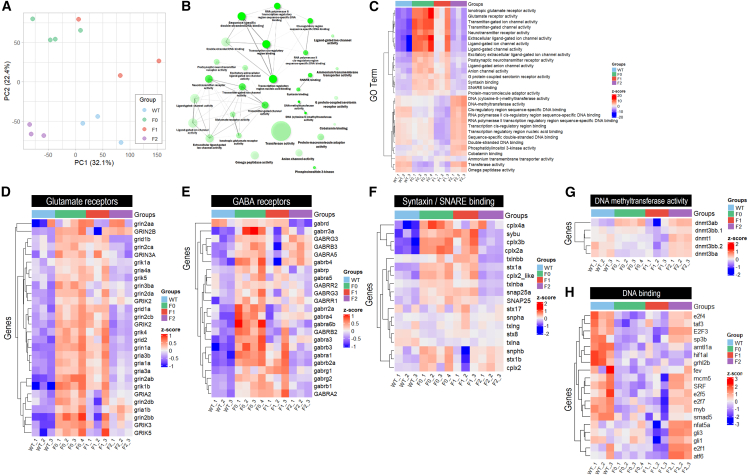
Transcriptomic analysis of marine medaka (*O. melastigma*) embryos at 5 dpf under control and elevated CO_2_-induced acidification across multiple generations (A) Principal-component analysis (PCA) of RNA sequencing data from 5 dpf embryos, comparing control (WT) and successive generations (F0, F1, and F2) exposed to acidified conditions. (B) Gene ontology (GO) enrichment analysis using parametric gene set enrichment analysis (PGSEA) on GO molecular function gene sets. The network plot displays these pathways, grouped based on gene expression levels and their correlations. Links between pathways indicate a 30% or greater shared gene composition, while nodes with more intense colors represent more significantly enriched gene sets and larger nodes denote more extensive gene sets. The analysis reveals key pathways affected by increased CO_2_-induced acidified conditions, including extracellular ligand-gated ion channel activities, synaptic vesicle transport mechanisms, DNA methylation processes, modulation of transcription initiation through targeted protein-DNA interactions at promoters, enhancers and related *cis*-regulatory modules. (C) Heatmap accompanied by an evolutionary tree, detailing the expression profiles of the top 30 pathways identified by GO analysis, indicating evolutionary relationships among genes. (D–H) Heatmaps present the expression pattern of genes (normalized as *Z* score) within specific functional categories across different generations: (D) glutamate receptors, (E) GABA receptors, (F) Syntaxin/SNARE binding proteins, (G) DNA (cytosine-5)-methyltransferases, and (H) DNA-binding activity. The tree of each heatmap clusters the genes based on the expression pattern similarity among groups.

In the early developmental stages of marine fish, the transcriptomic landscape of 5 dpf embryos across multiple generations was meticulously dissected using RNA sequencing and PCA (Figure 2A). This analytical method effectively differentiated between control groups and those exposed to acidified conditions, underscoring the value of comprehensive transcriptome analysis in pinpointing environmental stress responses. Subsequent in-depth gene ontology (GO) molecular function analysis of our dataset revealed the extensive impact of acidification stress on key shared significantly enriched gene sets in particular biological networks (Figure 2B). Notably, these networks were related to ligand-gated ion channel activities, synaptic vesicle transport mechanisms, modulation of transcription initiation through targeted protein-DNA interactions at promoters, enhancers, and related *cis*-regulatory modules, as well as DNA methylation processes (Figure 2B).

Comparative transcriptomics analysis of the top 30 pathways identified by GO analysis revealed pronounced alterations across generations in pathways associated with ligand-gated ion channel activity, glutamate and neurotransmitter receptors, anion channels, and Syntaxin/SNARE binding (Figure 2C). These pathways were significantly upregulated in the F0 and F1 generations but then recovered to levels similar to the wild-types in the F2 generation. Heatmaps presenting the expression patterns of genes within glutamate receptors (Figure 2D), GABA receptors (Figure 2E), and syntaxin/SNARE binding proteins (Figure 2F) across different generations further illustrated these changes. As ligand-gated channels mediate excitatory and inhibitory neurotransmission, their significant divergence in F0 and F1 embryos compared to wild-types indicated disrupted fast synaptic signaling. However, expression profiles in F2 embryos resembled wild-types, marking attenuated neurosignaling defects (Figures 2D and 2E). SNAREs enable synaptic vesicle fusion, and the observed changes in their expression suggested an elaborate neurophysiological response to counter transmission impairments induced by acidification.

Concurrently, the expression patterns of DNA (cytosine-5)-methyltransferases (*dnmt1* and *dnmt3*), crucial for DNA methylation, showed a decline in the F1 generation followed by an increase in the F2 generation (Figure 2G). These epigenetic variations suggested DNA modifications potentially driving adaptive signaling changes, bolstering the resilience of marine teleosts to environmental acidification. The substantial transcriptome changes demonstrated that acidification interfered with the complex regulation of neuronal activities, homeostasis, and epigenetic processes in early embryos exposed to prolonged CO_2_ stress.

Our transcriptomic analysis revealed a downregulation of genes involved in protein-DNA interactions at promoters, enhancers, and *cis*-regulatory modules, such as the E2F and GLI families, in the F0 and F1 generations compared to the wild-type group, suggesting a decreased regulation of transcription initiation in response to acidification stress. However, the expression levels of these genes returned to levels comparable to the wild-types in the F2 generation, indicating a potential multigenerational adaptive response (Figure 2H). Protein-DNA interactions at promoters, enhancers, and *cis*-regulatory modules regulate gene expression by controlling the binding of transcription factors to specific DNA sequences, which can profoundly impact the overall DNA methylation patterns in the genome and influence the expression of genes involved in various biological processes, including neurotransmission and epigenetic regulation. The recovery of gene expression levels in the F2 generation suggested that multigenerational exposure to acidified conditions may have induced heritable epigenetic changes that confer increased resilience to the disruptive effects of acidification stress. These findings highlight the potential for multigenerational resilience to environmental acidification through the modulation of transcriptional and epigenetic mechanisms in fish embryos.

### Ionic homeostasis and epigenetic inheritance for multigenerational adaptation to acidification in marine fish embryos

Our transcriptomic analysis revealed a significant decrease in the expression of genes related to ammonium transmembrane transporter activity in the F0 generation exposed to elevated CO_2_ conditions, followed by a recovery in the F1 generation (Figures 2B and 2C). These changes in gene expression were consistent with the observed multigenerational shifts in embryonic ammonium excretion rates (Figure 1D), suggesting a modulation of NH_4_^+^ excretion mechanisms, which play a crucial role in maintaining acid-base balance in fish through H^+^ excretion. To establish a precise physiological response indicator specifically targeting embryonic epithelial tissues, we employed *in vivo* scanning ion-selective electrode technique (SIET) assessments. These assessments demonstrated that all groups exposed to acidified conditions consistently exhibited increased H^+^ excretion from the yolk sac skin compared to wild-type controls, indicating an ongoing adaptive H^+^ secretion gradient in response to elevated CO_2_ levels (Figure S2; Table S4). This gradient is established and maintained by the coordinated action of essential transepithelial ion channels and/or regulators, which play a crucial role in regulating cellular pH homeostasis in response to acidification.

**Figure 3:**
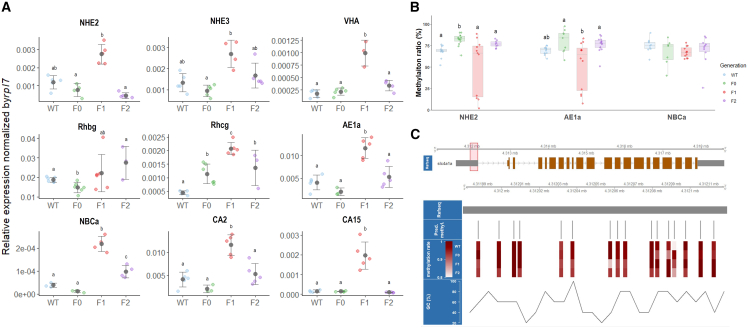
Transcriptional levels and epigenetic modifications of acid-base regulation genes in 5 dpf marine medaka (*O. melastigma*) embryos exposed to elevated CO_2_-induced acidification across multiple generations (A) Relative mRNA expression levels of key genes involved in H^+^ efflux (NHE2, NHE3, and VHA), NH_4_^+^ excretion (Rhbg and Rhcg), and HCO_3_^−^ secretion (AE1a, NBCa, CA2, and CA15), in marine medaka embryos at 5 dpf across generations (WT, F0, F1, and F2). CO_2_-acclimated F1 embryos exhibited significant up-regulation of these genes compared to other generations, while expression levels in CO_2_-acclimated F2 embryos were comparable to those of the wild-type (WT). Data are presented as mean ± SD (n = 4–5 per group). Statistical analysis followed a systematic approach: Shapiro-Wilk test for normality, followed by either one-way ANOVA with Tukey’s post-hoc test (normally distributed data) or Kruskal-Wallis test with appropriate post-hoc comparisons (non-normally distributed data). Different letters indicate significant differences between groups (*p* < 0.05). Complete statistical analyses are provided in Table S5. (B) DNA methylation levels at the promoter regions of selected acid-base regulation genes (NHE2, AE1a, and NBCa) in 5 dpf embryos across generations (WT, F0, F1, and F2). CO_2_-acclimated F1 embryos displayed significantly reduced methylation levels compared to other generations, indicating generation-specific epigenetic modifications in response to elevated CO_2_ conditions. Data are presented as mean ± SD (n = 9–14 per group). Statistical analysis followed a systematic approach: Shapiro-Wilk test for normality, followed by either one-way ANOVA with Tukey’s post-hoc test (normally distributed data) or Kruskal-Wallis test with appropriate post-hoc comparisons (non-normally distributed data). Different letters indicate significant differences between groups (*p* < 0.05). Complete statistical analyses are provided in Table S6. (C) Detailed examination of the DNA methylation rate at a specific upstream region of AE1a (slc4a1a), containing 16 CpG sites (predicted by the MethPrimer online software), in 5 dpf embryos across generations (WT, F0, F1, and F2). The color gradient (red: 100% methylation; white: 0% methylation) illustrates the variations in methylation across these sites, highlighting potential regions critical for epigenetic adaptation to acidified conditions. The schematic representation of the AE1a gene structure depicts the location of the analyzed CpG island relative to the transcription start site.

To elucidate the molecular mechanisms underlying these adaptive responses focused on acid-base and ion homeostasis, we examined the expression of those regulatory genes in 5 dpf embryos (Figure 3A; Table S5). This analysis revealed a significant upregulation of specific transporters governing key facets of epithelial ion movement in the F1 generation, including those involved in H^+^ efflux (NHE2, NHE3, and VHA), NH_4_^+^ excretion (Rhbg and Rhcg), and HCO_3_^−^ secretion and regulation (AE1a, NBCa, CA2, and CA15). The coordinated upregulation of these transporters in the F1 generation suggests a concerted effort to enhance the capacity for H^+^ secretion and acid-base regulation in response to acidification stress. Intriguingly, this acidification-responsive elevation across interconnected regulatory nodes rebounded back toward homeostasis in the F2 generation, suggesting an augmented resilience to acid-base perturbations in the multigenerationally exposed progeny. The ability of the F2 generation to maintain cellular pH homeostasis more effectively than the F1 generation may be attributed to the fine-tuned regulation of these essential ion channels and their associated transporters, which have likely undergone multigenerational adaptation in response to prolonged exposure to acidified conditions.

To investigate the potential role of epigenetic modifications, specifically DNA methylation, in facilitating multigenerational acclimation, we examined methylation profiles at the promoter regions of key transepithelial acid-base regulatory genes located on ionocytes, including NHE2,^38^ AE1a,^39^ and NBCa.^40^ Methylated DNA immunoprecipitation (MeDIP) was employed to assess differential methylation patterns across generations. This targeted analysis aimed to validate the hypothesis that alterations in DNA methylation status at these critical gene regulatory regions contribute to the adaptive responses and resilience observed in the multigenerationally exposed progeny. By elucidating the potential role of methylation-mediated epigenetic reprogramming in modulating the expression of these essential homeostatic regulators, our findings showed that, except for a notable alteration at the AE1a promoter in testes (Figure S3), parental analyses showed negligible generational methylation deviations. However, a significant reduction in DNA methylation levels at the promoter regions of two critical acid-base regulatory genes in ionocytes, NHE2 and AE1a, emerged in F1 5 dpf embryos before partially recovering in F2 (Figure 3B; Table S6). In teleosts, changes in promoter DNA methylation have been demonstrated to be associated with alterations in gene transcription,^41^^,^^42^ the observed hypomethylation events at the promoter regions of crucial acid-base regulatory genes may enable the adaptive regulation of these key transporters. To gain a more comprehensive understanding of the epigenetic landscape, we conducted a detailed analysis of the AE1a promoter region. This investigation revealed a substantial increase in hypomethylated CpG sites within the 4.312037 mb–4.312065 mb region in both the F1 and F2 generations (Figures 3C and S4). The observed hypomethylation pattern in specific CpG sites within the promoter region of AE1a (Figures 3C and S3) correlates with the increased transcriptional activity of this gene (Figure 3A), suggesting an epigenetically driven enhancement of its function in regulating anion transport at the basolateral surface of epithelial cells. This targeted epigenetic modulation likely contributes to the improved ability of fish embryos to maintain ionic balance when faced with the persistent challenges posed by CO_2_-induced acidification. The multigenerational persistence of this hypomethylation pattern and its associated transcriptional upregulation in the F2 generation highlights the potential role of epigenetic mechanisms in facilitating the adaptive response to ocean acidification and the maintenance of physiological homeostasis across multiple generations.

## Discussion

This study presents robust empirical evidence concerning physiological plasticity and compensatory strategies in marine medaka subjected to continuous ocean acidification across multiple generations. Our findings illustrate that elevated CO_2_ instigates intergenerational reprogramming of essential osmoregulatory and acid-base regulatory mechanisms. Although our experimental framework facilitated a direct assessment of cumulative multigenerational responses, we acknowledge that it employs a common reference-point design wherein all CO_2_-exposed generations (F0, F1, and F2) are compared against a shared WT baseline. This methodology was purposefully selected to ensure biological consistency and interpretability, as the maintenance of a single reference group minimizes potential genetic or physiological drift in untreated lineages that may otherwise obscure acidification-specific effects. We recognize that this design precludes generation-matched control comparisons, a limitation that necessitates careful consideration when interpreting our results. Nevertheless, the phenotypic and transcriptomic profiles revealed coherent and repeatable shifts in response to elevated CO_2_ that are consistent with established acid-base regulatory mechanisms. The progressive nature of these changes across generations suggests directional adaptive progression rather than random physiological drift. Through systematic replication and comprehensive analyses, we were able to precisely distinguish between treatment effects and generational variation, thereby providing robust evidence for acidification-induced adaptations. We have identified distinct generational patterns of adaptation to elevated CO_2_ levels through a comprehensive analysis of developmental, physiological, and molecular responses. The physiological homeostasis and gene expression of F0 embryos were disrupted, while F1 embryos exhibited compensatory molecular responses and significant epigenetic modifications. F2 embryos exhibited evidence of adaptive resilience. These progressive phenotypic and molecular adjustments are driven by sustained environmental pressure rather than temporal variation, as confirmed by the control parameters’ consistency throughout the experimental period, in contrast with the distinct treatment-specific response patterns across generations. Although field studies establish ecological context, our controlled experimental approach facilitated mechanistic assessment from molecular to organismal scales,^43^ revealing how coordinated changes in gene expression and epigenetic modifications facilitate adaptive responses to ocean acidification.^44^^,^^45^

Our thorough assessments demonstrate that significant physiological processes have changes unique to each generation. In F0 embryos, acidification disrupted ionic regulation and developmental trajectories, resulting in altered metabolic rates and cardiac function. However, compensatory responses were observed in subsequent generations. F1 embryos exhibited improved acid-base regulatory capacity by upregulating specific ion transporters, while F2 embryos partially regained physiological homeostasis. These generation-specific responses align with but extend beyond previous observations in other marine teleosts. For instance, while Japanese medaka (*Oryzias latipes*) showed pronounced embryonic sensitivity to acidification,^24^ and Atlantic cod (*Gadus morhua*) exhibited compensatory upregulation of acid-base regulatory mechanisms,^46^ our study reveals how such adaptations evolve across multiple generations. The enhanced resilience in F2 embryos, which we observed as a result of the progressive refinement of physiological responses, indicated that marine teleosts may have a greater capacity for multigenerational adaptation than was previously recognized. However, growth and development were still being influenced by sustained exposure to acidification.

The multigenerational analysis identified specific metabolic signatures that suggested a progressive adaptation to acidified conditions. The metabolic responses observed across generations highlight specific energy allocation strategies. F0 embryos showed impaired ammonium excretion and altered oxygen consumption patterns, suggesting initial physiological stress, while subsequent generations demonstrated more stable metabolic profiles. Notably, the Bohr effect manifested through declining heartbeat rates in F2 embryos,^47^^,^^48^ indicating persistent pH-dependent impacts on oxygen transport efficiency despite other adaptive improvements. These metabolic adjustments appeared coordinated with broader physiological responses, particularly acid-base regulation. The generation-specific patterns we observed in ion transport and metabolic pathways suggested that maintaining acid-base homeostasis under chronic acidification required substantial energetic investments, even as compensatory mechanisms developed across generations. This metabolic reconfiguration likely represents a crucial component of multigenerational adaptation, though it may come at the cost of reduced growth efficiency, as evidenced by the smaller size of F1 and F2 embryos. Such trade-offs between homeostatic maintenance and development provides important insights into the energetic constraints facing marine organisms under sustained environmental stress.

Through omics technologies, the intricacies of molecular pathways came into focus, revealing a fundamental principle of resilience: the capacity to harness the pressure of external demands as a catalyst for internal adaptation. The fully sequenced marine medaka genome represents a pivotal advancement for elucidating marine teleost adaptations, integrating theoretical frameworks with applied investigations.^49^^,^^50^ Our multigenerational transcriptomic analyses of early life stages permitted systemic insight into acidification responses over prolonged exposures. PCA revealed significant generational alterations in RNA expression associated with ion channel and transport machinery, which are vital for homeostasis across epithelial and neural matrices. Relative to wild-types, the F1 generation displayed elevated transcription before F2 stabilization, implying later generational realignment given incomplete ionic/acid-base compensation. Hypercapnia can severely disrupt neurosensory development and behaviors in susceptible larvae.^51^ Disruptions to GABAergic signaling, a crucial component of nervous system regulation, may stem from alterations in ionic gradients and impaired inhibitory control over excitatory neurotransmitters such as glutamate.^40^^,^^52^ While anxiety-related behavioral abnormalities were anticipated, our study found upregulations in neurotransmitter receptors solely in F0 and F1 medaka, with F2 embryos exhibiting expressions similar to wild-types. The dampened transmission signaling alterations in F2 embryos indicated an enhanced adaptive resilience in preserving neurosignaling functions despite prolonged CO_2_ exposure, suggesting that multigenerational exposure to acidified conditions may have triggered adaptive mechanisms to maintain proper neurotransmission under environmental stress. This observed recovery indicated that the presumed long-lasting neuronal vulnerabilities resulting from even brief acidification exposures may not be universally applicable.^53^^,^^54^ However, it is important to note that academic misconduct has compromised the integrity of several foundational studies in this field,^55^^,^^56^ and a general “decline effect” among reported responses indicates that some earlier findings may contain outliers or misleading data. Despite these setbacks, further investigations into the developmental consequences of GABAergic and interneuron signaling perturbations remain critical to advancing our understanding of the impacts of acidification on neurological development and function. Lengthier intergenerational studies across cellular and organismal strata are vital. Integrative models like the medaka, with well-annotated genomes and established multigenerational rearing frameworks, offer resonant platforms to advance physiological frontiers and inform conservation efforts.

Our experimental design elucidates the molecular mechanisms of adaptive responses, providing significant insights into inheritance patterns during prolonged environmental stress. Our study reveals significant physiological and epigenetic alterations across generations due to prolonged CO_2_ exposure; however, these results should be understood within the framework of multigenerational rather than transgenerational effects. RNA-seq analysis demonstrated increased expression of DNA (cytosine-5) methyltransferases, indicating a significant advancement in the comprehension of epigenetic regulation in response to acidification. Distinct DNA methylation signatures associated with specific generations were identified in acid-base regulatory genes, with F1 embryos showing coordinated alterations in both methylation status and the expression patterns of DNA methyltransferases. Epigenetic modifications were particularly evident in the hypomethylation of key epithelial ion transport genes NHE2 and AE1a in 5 dpf embryos, which corresponded with increased transcriptional activation of acid-base regulatory pathways. An analysis of dimorphic DNA methylation patterns in the AE1a promoter region CpG island identified regulatory mechanisms that connect environmental perturbation to gene expression modulation. The establishment of quantitative relationships among the methylation status of specific regulatory regions, their corresponding expression levels, and resultant physiological adaptations offer mechanistic insights into epigenetic regulation in the context of ocean acidification. Our study integrates methylation profiles with transcriptional dynamics and physiological responses, demonstrating the role of epigenetic modifications in orchestrating adaptive responses under acidified conditions. The relationship among promoter methylation patterns, transcriptional activity, and acid-base regulatory capacity indicated that these modifications were essential molecular mechanisms for sustaining physiological homeostasis during chronic acidification stress. The transmission mechanisms of epigenetic modifications across generations are not yet fully understood, especially concerning their stability and inheritance patterns.^57^^,^^58^ However, our findings indicated their essential role in allowing early life stages of teleosts to maintain acid-base equilibrium during significant acidification. This molecular framework offers essential insights into adaptation mechanisms; however, it is important to note that these responses arise under continuous exposure conditions rather than indicating transgenerational inheritance.

Adaptability, genetic bases, and epigenetic refinement are pivotal in evolutionary storytelling.^59^^,^^60^ They weave the biota of Earth,^61^ requiring adjustments in energy flow to build physiological anchors against environmental threats.^62^^,^^63^ Even though epigenetic dynamics are essential to the evolutionary process, they have also been demonstrated to be associated with cellular energy metabolism^64^ and organismal fitness.^65^ The growing danger of ocean acidification drives marine life to walk a tightrope of energy reallocation, balancing adaptability with survival imperatives.^66^^,^^67^ This tapestry of marine history may represent two different but related scenarios: the extinction of some species and the emergence of others.^68^^,^^69^ To make sense of this dynamic marine story, it is necessary to integrate the knowledge of energy paradigms, adaptive counterbalances, and epigenetic revolutions. An extensive molecular perspective is required to properly anticipate the future rhythms of marine species undergoing re-adjustment. Our discoveries enhance the marine molecular dialectic in the face of acidification, illuminating the intricate dance of adaptation and resilience, thereby enhancing our arsenal against the different ecological onslaughts.

### Limitations of the study

This study has several important limitations worth noting. First, our experimental design employed a common reference-point comparison where all CO_2_-exposed generations (F0, F1, and F2) were compared against a single wild-type control rather than generation-matched controls, which may limit our ability to distinguish treatment effects from potential generational drift. Second, the research was conducted under strictly controlled laboratory conditions utilizing a singular marine fish species (*O*. *melastigma*), which may not comprehensively encapsulate the intricate and diverse marine environment wherein multiple stressors concurrently interact. This limitation may impede the generalizability of the findings to other marine taxa. Third, our findings represent multigenerational rather than transgenerational effects, as embryos were continuously exposed to acidified conditions rather than inheriting stable epigenetic modifications from unexposed parents. Fourth, while we have identified epigenetic modifications correlated with alterations in gene expression, the mechanisms governing the inheritance and stability of these modifications across generations necessitate further inquiry. Finally, our assessment was limited to early life stages (up to 7 dpf), and longer-term effects on adult fitness, reproduction, and population-level consequences remain to be determined.

## Resource availability

### Lead contact

Further information and requests for resources and reagents should be directed to and will be fulfilled by the lead contact, Yung-Che Tseng (yctseng@gate.sinica.edu.tw).

### Materials availability

This study did not generate new unique reagents, cell lines, or genetically modified organisms. All chemical reagents, commercial assays, and laboratory equipment used in this study are commercially available from the sources listed in the key resources table. Marine medaka (*O. melastigma*) fish stocks used in this study are maintained at the Marine Research Station, Institute of Cellular and Organismic Biology, Academia Sinica, Taiwan. These fish stocks are available from the lead contact upon reasonable request, subject to appropriate institutional material transfer agreements and compliance with local regulations for marine organism transfer.

### Data and code availability

• All raw RNA sequencing data generated in this study have been deposited in the NCBI Sequence Read Archive (SRA) and are publicly available under BioProject: PRJNA1086368; BioSample: SAMN40375180.
• All other raw data supporting the findings of this study, including physiological measurements (oxygen consumption, NH_4_^+^ excretion, heartbeat frequency), morphometric data, scanning ion-selective electrode technique (SIET) measurements, gene expression data (qRT-PCR), DNA methylation data (MeDIP and bisulfite sequencing), and processed transcriptomic datasets, have been deposited in the Dryad Digital Repository and are publicly available at https://doi.org/10.5061/dryad.4xgxd25j7.
• This paper does not report original code or novel algorithms. All data analysis was performed using publicly available software as detailed in the STAR Methods. Custom analysis scripts for data processing and statistical analysis are available from the lead contact upon reasonable request.
• Any additional information required to reanalyze the data reported in this paper is available from the lead contact upon request.

## Acknowledgments

The authors express their appreciation for the Marine Research Station (ICOB, Academia Sinica) for supporting multigenerational rearing and caring for marine medaka during the experiments. We would also like to thank Academia Sinica’s NGS Core for their assistance in our transcriptomic experiments and for providing their sequencing methodology.

Y.-C.T. was financially supported by grants from the Academia Sinica, Taiwan (AS-TP-109-L02) and the National Science and Technology Council (NSTC), Taiwan (NSTC 114-2313-B-001-007-MY3).

## Author contributions

T.-Y.L. and Y.-C.T. wrote the manuscript. T.-Y.L., J.-J.Y., and O.H. processed the transcriptomic data. T.-Y.L., L.-Y.L., and Y.-J.G. performed the development and physiological assessments. T.-Y.L. and G.-C.W. performed the epigenetic assays. G.-C.W., M.-T.C., P.-P.H., and Y.-C.T. designed the study. Y.-C.T. secured funding for the study. All authors editorially contributed to the manuscript.

## Declaration of interests

The authors declare no competing interests.

## STAR★Methods

### Key resources table

**Table:** 

REAGENT or RESOURCE	SOURCE	IDENTIFIER
**Antibodies**		

Anti-5-methylcytosine monoclonal antibody	EpiGentek	Cat# A-1014

**Biological samples**		

Marine medaka (*Oryzias melastigma*) embryo	City University of Hong Kong (Dr. Doris W.T. AU)	N/A

**Chemicals, peptides, and recombinant proteins**		

QIAzol Lysis Reagent	QIAGEN	Cat# 79306
SuperScript III Reverse Transcriptase	Invitrogen	Cat# 18080044
SYBR Green I Master Mix	Roche	Cat# 04887352001
MS-222 (Tricaine methanesulfonate)	Sigma-Aldrich	Cat# A5040
Dimethyl chlorosilane	Sigma-Aldrich	Cat# 85126
H^+^ ionophore I cocktail B	Sigma-Aldrich	Cat# 95297

**Critical commercial assays**		

RNeasy Plus Universal Mini Kit	QIAGEN	Cat# 73404
EpiQuik Tissue Methylated DNA Immunoprecipitation Kit	EpiGentek	N/A
EZ DNA Methylation Kit	ZYMO Research	Cat# D5001
Wizard Genomic DNA Purification Kit	Promega	Cat# A1120
Agilent RNA 6000 Nano Kit	Agilent Technologies	Cat# 5067-1511

**Deposited data**		

Raw RNA sequencing data	This paper	NCBI SRA: BioProject PRJNA1086368 (https://www.ncbi.nlm.nih.gov/bioproject/PRJNA1086368); BioSample: SAMN40375180
Processed data and additional raw data	This paper	Dryad Digital Repository: https://doi.org/10.5061/dryad.4xgxd25j7
Marine medaka genome reference	Ensembl Genome Browser	Ensembl: Om_v0.7.RACA

**Experimental models: Organisms/strains**		

*Oryzias melastigma*	Dr. Doris W. T. AU of the Department of Biology and Chemistry at the City University of Hong Kong	RRID: NCBITaxon_30732

**Oligonucleotides**		

qRT-PCR primers: See Table S8	This paper	Table S8
AE1a bisulfite forward primer	This paper	5'-TTTAGTGATTAATGGGATTTTAGAA-3'
AE1a bisulfite reverse primer	This paper	5'-AAAAACCACACCCCTACCTATATAC-3'
MeDIP primers: See Table S9	This paper	Table S9

**Software and algorithms**		

Fiji/ImageJ	N/A	https://imagej.net/software/fiji/
CLC Genomics Workbench: version 20.0	CLC Bio-Qiagen, Denmark	https://digitalinsights.qiagen.com/products-overview/discovery-insights-portfolio/analysis-and-visualization/qiagen-clc-genomics-workbench/
R-based integrated Differential Expression and Pathway (iDEP.96)	N/A	http://bioinformatics.sdstate.edu/idep96/
GraphPad	GraphPad Software, United States	https://www.graphpad.com/
FastQC quality control tool	Babraham Bioinformatics	v.0.11.9; RRID:SCR_014583
CO2SYS calculation software	Lewis and Wallace (1998)^70^	N/A
MethPrimer	N/A	http://www.urogene.org/methprimer/
R: version 4.4.1	R Core Team	https://www.r-project.org/

**Other**		

NovaSeq 6000 sequencing	Genomics Biotech Co., Taipei, Taiwan	Illumina NovaSeq 6000 platform
STM800 StereoZoom Microscope	BEL Engineering, Italy	Model STM800
Automated pH control system	Macro Taiwan	Model E-M03
LightCycler 480 Real-Time PCR System	Roche	Cat# 05015243001
Agilent 2100 Bioanalyzer	Agilent Technologies	Model G2939BA
SpectraMax i3 Multi-Mode Microplate Reader	Molecular Devices	Model i3x
OXY-4 mini fiber optic oxygen transmitter	PreSens Precision Sensing	Model OXY-4 mini
pH electrode SenTix 41	WTW, Germany	Cat# 103422
pH meter	WTW, Germany	Model pH 3310
Sony NEX-6 digital camera	Sony	Model NEX-6

### Experimental model and study participant details

#### Marine medaka maintenance and ethics

Marine medaka (*O. melastigma*) were originally provided by Dr. Doris W. T. AU of the Department of Biology and Chemistry at the City University of Hong Kong in 2015. All experimental procedures were approved by the Academia Sinica Institutional Animal Care and Biosafety Committee (approval no. BSF0419-00004071) and conducted in accordance with institutional guidelines for animal welfare.

To minimize environmental variation effects and ensure genetic stability, medaka were acclimated and bred in a natural seawater flow-through system at the Marine Research Station (Institute of Cellular and Organismic Biology, Academia Sinica, Taiwan) for 6 complete generations (>4 years) before experimental manipulation. This extended acclimation period established stable baseline conditions essential for multigenerational studies.

#### Experimental design

This study employed a multigenerational experimental design comparing marine medaka embryos across four groups: wildtype controls (WT, pH 8.1) and three successive generations exposed to acidified conditions (F0, F1, F2, pH 7.6) (Figure 1A). Both male and female fish were used throughout the study, with sex ratios maintained at approximately 1:1 in all breeding tanks. Natural seawater was pumped from offshore areas around Yilan County, Taiwan, with baseline pH values ranging 8.114 ± 0.014. Fish were maintained under standardized conditions and provided with commercial artificial food three times daily (TB55S, Hai Feng Feeds Co., LTD, Taiwan; composition: crude protein ≥45%, crude fat ≥6%, crude ash ≤16%, moisture ≤8%).

### Method details

#### Seawater chemistry control and monitoring

Two distinct water conditions were maintained throughout the study: control tanks (WT, pH 8.1, pCO_2_ ∼ 0.03 kPa) and acidified tanks (pH 7.6, pCO_2_ ∼ 0.14 kPa). An automated pH detection and control system (E-M03, Macro, Taiwan) equipped with calibrated pH electrodes (accuracy ±0.01 pH units) continuously monitored seawater pH. The system regulated CO_2_ injection through a feedback-controlled solenoid valve to maintain target pH levels. pH electrodes were calibrated weekly using NBS standard buffers (pH 4.01, 7.00, and 10.01). Each experimental condition was replicated in four independent tanks (300 L each) containing approximately 30 adult fish with a 1:1 male:female sex ratio. Seawater conditions were maintained as follows: salinity 31.73 ± 1.79‰, temperature 27.89 ± 0.79°C, photoperiod 10 h dark/14 h light cycle. Water chemistry parameters were monitored according to the following schedule, and detailed seawater physicochemical parameters are provided in Table S7:

• **Daily monitoring**: pH and temperature using a calibrated pH meter (pH 3310, WTW, Germany) with pH electrode (SenTix® 41, WTW, Germany)
• **Weekly monitoring**: Total alkalinity (TA) measured following the spectroscopic method of Sarazin et al. (1999),^71^ and dissolved inorganic carbon (DIC) using an automatic infrared analyzer (AS-C3, Apollo SciTech, USA) with quantification via non-dispersive infrared CO_2_/H_2_O analyzer (LI-7000, LI-COR, USA)
• **Calculated parameters**: pCO_2_ values calculated using CO2SYS software module.^70^

#### Sample collection strategy

The eggs and embryos utilized for all analyses were randomly collected from the replicate tanks to ensure biological and environmental replication. In conducting all experimental measurements, a systematic sampling approach was employed, whereby each statistical replicate (n) comprised a pooled sample containing embryos drawn equally from each of the four independent tanks, thereby mitigating potential tank-specific effects. For instance, a single replicate for metabolic rate measurements consisted of a pooled sample of 60 embryos (15 embryos per tank × 4 tanks). This stratified sampling design ensured that each replicate accurately represented the entire experimental population while providing an adequate amount of biological material for precise measurements. These fertilized eggs were raised in both settings and cultured into adult individuals (F0), after which previous phases were repeated to continue rearing and breeding for two further generations (F1 and F2) (Figure 1A). The experimental protocols were approved by the Academia Sinica Institutional Animal Care and Biosafety Committee (approval no. BSF0419-00004071).

#### Morphometric and developmental assessments

Growth parameters (interocular distance, eye diameter, body total length, body height, and body width) were measured from 2 to 7 days post-fertilization (dpf) using calibrated digital image analysis (Fiji/ImageJ software) with a stereo microscope (STM800 StereoZoom Microscope, BEL Engineering, Italy). Measurements were conducted on randomly selected individuals from control (WT, N=25) and acidified groups (F0, N=24; F1, N=10; F2, N=10) with calibrated scale bars for accurate quantification.

#### Metabolic rate measurements

Oxygen consumption and NH_4_^+^ excretion were measured at 5 dpf using closed-system respirometry following established protocols. Measurements were conducted on pooled groups of 60 embryos in sterile glass respirometry chambers (volume 2 mL) maintained at constant temperature (27.89 ± 0.1°C) in a temperature-controlled water bath.

##### Oxygen consumption

Oxygen consumption (μmol O_2_/h.g) was monitored continuously using calibrated fiber optic oxygen sensors (PreSens oxygen micro optode, type PSt3) connected to an OXY-4 mini transmitter (PreSens). Sensors were calibrated using air-saturated seawater (100%) and sodium sulfite-saturated water (0%). Parallel blank chambers without embryos were used to correct for background bacterial respiration.

##### Ammonium excretion

NH_4_^+^ excretion was determined using a modified described by Holmes et al.,^72^ water samples from each chamber were collected to determine the NH_4_^+^ excretion rate by eggs expressed as μmol NH_4_^+^/h.g. 25 μL of water samples were mixed with 100 μL of NH_4_^+^ assay working reagent in a 96-well black microplate and then read by a SpectraMax i3 (Molecular Devices, Danaher, US), with the wavelength of excitation and emission at 360 and 420 nm, after 2.5 hours of incubation at the room temperature.

#### Proton excretion measurements

The Scanning Ion-selective Electrode Technique (SIET) was employed to assess H^+^ excretion on the 5 dpf embryonic surface. Micropipettes with a tip diameter of 3-4 μm, prepared from capillary glass tubes (no. TW 150-3; World Precision Instruments, Sarasota, FL, USA), were first baked at 120°C overnight and then coated with dimethyl chlorosilane (Sigma-Aldrich) for 30 minutes. For constructing ion-selective microelectrodes, the micropipettes were backfilled with electrolytes (40 mM KH_2_PO_4_ and 15 mM K_2_HPO_4_) extending 1 cm and frontloaded with a 20-30 μm column of H^+^ ion-exchange cocktail (H^+^ ionophore I cocktail B, Sigma-Aldrich). Calibration involved measuring the Nernstian response of each microelectrode across standard solutions (pH 7, pH 8, pH 9), yielding a Nernstian slope of 58.6±0.8 (n=10) for H^+^. The procedure was conducted at room temperature in a sealed plastic chamber filled with the recording medium. The seawater (SW) recording medium composition was 350.9 mM NaCl, 45.7 mM MgCl_2_·6H_2_O, 24.2 mM Na_2_SO_4_, 8.9 mM CaCl_2_·2H2O, 7.8 mM KCl, 2 mM NaHCO_3_, 300 μM Tricine, and 0.3 mg/L ms222, adjusted to pH 8.0. Embryos were centered in the chamber, and voltages at the embryonic surface were recorded. Subsequently, the probe was moved ∼10 mm away from the embryo to record background H^+^ activity. The voltage differences (mV) between the target point and the background were then converted to ionic gradients (Δ[H^+^]).

#### Heartbeat frequency analysis

A 5 dpf medaka embryo was positioned under a STM800 StereoZoom Microscope (BEL Engineering, Italy). Using a Sony NEX-6 camera, one-minute videos focusing on the embryo's heart cavity were captured. A total of 10-12 individuals were analyzed in each group. These videos were analyzed using Fiji software, which extracted heart size values at a rate of 30 frames per second. The extracted data were then processed using a Fourier transform to accurately determine the heartbeat frequency over the one-minute recording period.

#### RNA-Seq analysis of medaka embryos

To assess the transcriptomic profiles of medaka embryos, a pooled group of 30 medaka embryos from each cohort was thoroughly homogenized using QIAzol® Lysis Reagent, part of the RNeasy Plus Universal Mini Kit by QIAGEN (Hilden, Germany), followed by the addition of gDNA Eliminator Solution to remove genomic DNA contamination. RNA extraction was performed according to the manufacturer's protocol. The quality and integrity of the extracted RNA samples were rigorously evaluated by Genomics (Taipei County, Taiwan) using the Agilent 2100 Bioanalyzer and the Agilent RNA 6000 Nano Kit (Agilent, California, United States). Upon passing the quality assessment, paired-end sequencing libraries with a read length of 150 bp were prepared and sequenced using the Illumina NovaSeq 6000 platform, with an expected data output of approximately 6 Gb per sample. This sequencing task was executed under the expertise of Genomics (Taipei, Taiwan). Following sequencing, the raw data underwent stringent quality control checks. The RNA-seq reads were trimmed and mapped to the *O. melastigma* reference genome (Om_v0.7.RACA, obtained from Ensembl) usinCLg the CLC Genomics Workbench (version 20.0, CLC Bio-Qiagen, Denmark). The RNA-seq profiling was uploaded as the SRA data (BioProject: PRJNA1086368; BioSample: SAMN40375180) and then used to compile gene expression profiles as well as perform comparative analyses.

Data analysis was conducted using the R-based integrated Differential Expression and Pathway (iDEP.96) analysis tool.^73^ This advanced analysis facilitated the identification and distinction of significantly altered Gene Ontology (GO) Molecular Function pathways among the experimental groups through Parametric Gene Set Enrichment Analysis (PGSEA), allowing for a comprehensive understanding of the transcriptional changes induced by the experimental conditions. Based on the pathway results, we selected genes that might be influenced by CO_2_ exposure and normalized their read counts using Z-scores. Z-scores were calculated gene-by-gene by subtracting the average gene abundance from the raw expression and dividing by the standard deviation (SD) to compare expression levels across samples. The obtained Z-scores were then utilized to create heatmaps.

#### Gene expression profiling

To investigate the expression of relevant genes, cDNA was synthesized from previously extracted RNA using SuperScript III Reverse Transcriptase (Invitrogen, Carlsbad, CA, USA). Subsequent quantitative real-time polymerase chain reaction (qRT-PCR) measurements of specific gene expressions were conducted using the Roche LightCycler® 480 System (Roche Applied Science, Mannheim, Germany), employing bespoke primers listed in Table S8. Standard curves were generated for each target gene to assess linearity and ensure the accuracy of the results. The ribosomal protein L7 (*rpl7*) gene from *O. melastigma* was used as a reference gene for normalization.^74^

#### DNA methylation level assessments

Genomic DNA (gDNA) was extracted from pooled embryos (5 dpf) using the Wizard® Genomic DNA Purification Kit (Promega, Wisconsin, USA). Each MeDIP sample (replicate) comprised a total of 20 embryos (5 embryos from each of the four tanks) to ensure even representation from all tanks. The purified DNA was then fragmented via sonication for subsequent Methylated DNA Immunoprecipitation (MeDIP) employing the EpiQuik™ Tissue Methylated DNA Immunoprecipitation Kit (Epigentek, New York, United States). Fragmented DNA (1 μg) was immunoprecipitated with an anti-5-methylcytosine antibody (1 μg) bound to each well of the assay plate. The IP DNA was collected, and the promoter methylation levels were determined by qPCR using specific primers (Table S9). Methylation levels were calculated using the formula:

IPDNA/InputDNA=2ˆ(ΔCt(Input−IP)).

For enhanced resolution of AE1a methylation differences among generations, 1 μg of gDNA was analyzed using the EZ DNA Methylation™ Kit (ZYMO Research, California, United States). Bisulfite-converted DNA was PCR amplified using bisulfite-treated DNA-specific primers (forward: 5'- TTTAGTGATTAATGGGATTTTAGAA-3'; reverse: 5'- AAAAACCACACCCCTACCTATATAC-3') targeting the AE1a promoter region (Om_v0.7.RACA:NVQA01000010.1:19437452-19437381), which was predicted using MethPrimer online software (Li and Dahiya, 2002). PCR products were sequenced by Genomics (Taipei, Taiwan) to determine unmethylated and methylated CpG sites. 16 sites within this CpG island were analyzed. The demethylation dimorphism of the AE1a promoter was presented as the percentage of CpG sites without methylation.

### Quantification and statistical analysis

#### Statistical design and approach

Statistical analyses were designed to assess multigenerational responses by comparing all CO_2_-exposed generations (F0, F1, F2) against the wildtype (WT) reference group, which served as the consistent baseline throughout the study. This common reference-point design was selected to ensure biological consistency and minimize potential genetic drift in control lineages that might otherwise obscure acidification-specific effects.

#### Statistical testing procedures

The data analysis was meticulously designed to monitor the multigenerational responses to ocean acidification by comparing all CO_2_-exposed generations (F0, F1, F2) to the consistent wildtype (WT) reference group, which served as the foundational baseline. This approach enabled the assessment of cumulative multigenerational effects and adaptive trajectories across generations. The Shapiro-Wilk test was initially used to assess data normality using the Normality and Lognormality Tests in Prism (GraphPad Software). Levene's test was applied to evaluate the homogeneity of variances for datasets intended for ANOVA analysis, using the leveneTest() function from the car package in R.^75^ For normally distributed data with homogeneous variances, one-way ANOVA with Tukey's post-hoc test was employed in Prism (GraphPad Software). Welch’s ANOVA (the oneway.test() function from R’s base stats package^76^) was used to assess group differences in normally distributed data with heterogeneous variances, as indicated by Levene’s test, followed by the Games-Howell post-hoc test using the rstatix package.^77^ The Kruskal-Wallis test, along with appropriate post-hoc comparisons, was conducted for non-normally distributed data in Prism (GraphPad Software). Results are reported as mean ± standard deviation (SD). Supplemental tables encompass comprehensive statistical outputs, providing direct comparisons of each acidification-exposed generation to the WT baseline. All analyses were performed using GraphPad Software and R.

## Supplemental information

Document S1. Figures S1–S4

Table S1. Growth parameters, including interocular distance, eye diameter, body total length, body height, and body width of 5 dpf marine medaka embryos across multiple generations under control and CO_2_-acidified conditions

Table S2. Metabolic features, including oxygen consumption and ammonium excretion rates of 5 dpf marine medaka embryos across transgenerational CO_2_ exposure

Table S3. Heartbeat frequency measurements of 5 dpf marine medaka embryos showing cardiovascular responses to multigenerational acidification

Table S4. Proton excretion rates from yolk sac epithelium of 5 dpf marine medaka embryos demonstrating acid-base regulatory capacity under CO_2_ stress

Table S5. Gene expression analysis of acid-base regulatory genes in 5 dpf marine medaka embryos across four generations (A) *slc9a2* (NHE2); (B) *slc9a3.2* (NHE3); (C) *atp6v1a* (VHA); (D) *slc4a1a* (AE1a); (E) *slc4a4a* (NBCa); (F) *CA2*; (G) *CA15*; (H) *rhbg*; (I) *rhcg*.

Table S6. DNA methylation analysis by methylated DNA immunoprecipitation (MeDIP) of acid-base regulatory gene promoters in 5 dpf marine medaka embryos (A) *slc9a2* (NHE2); (B) *slc4a1a* (AE1a); (C) *slc4a4a* (NBCa).

Table S7. Physicochemical parameters of experimental seawater including pH, temperature, salinity, pCO_2_, total dissolved inorganic carbon (C_T_), and total alkalinity (A_T_)

Table S8. Primer sequences for quantitative RT-PCR analysis of acid-base regulatory genes with amplicon sizes and accession numbers

Table S9. Primer sequences for quantitative RT-PCR analysis of promoter methylation levels in immunoprecipitated DNA samples

## References

[1] Paris J.D., Riandet A., Bourtsoukidis E., Delmotte M., Berchet A., Williams J., Ernle L., Tadic I., Harder H., Lelieveld J. (2021). Shipborne measurements of methane and carbon dioxide in the Middle East and Mediterranean areas and the contribution from oil and gas emissions. Atmos. Chem. Phys..

[2] Revelle R., Suess H.E. (1957). Carbon dioxide exchange between atmosphere and ocean and the question of an increase of atmospheric CO_2_ during the past decades. Tellus.

[3] Tanhua T., Keeling R.F. (2012). Changes in column inventories of carbon and oxygen in the Atlantic Ocean. Biogeosciences.

[4] Bao Y., Qiao F., Song Z. (2012). Historical simulation and twenty-first century prediction of oceanic CO_2_ sink and pH change. Acta Oceanol. Sin..

[5] Williamson P., Turley C. (2012). Ocean acidification in a geoengineering context. Philos. Trans. R. Soc., A.

[6] Rafiee A., Khalilpour K.R., Milani D., Panahi M. (2018). Trends in CO_2_ conversion and utilization: A review from process systems perspective. J. Environ. Chem. Eng..

[7] Kelly M.W., Hofmann G.E. (2013). Adaptation and the physiology of ocean acidification. Funct. Ecol..

[8] Stillman J.H., Paganini A.W. (2015). Biochemical adaptation to ocean acidification. J. Exp. Biol..

[9] Wang X., Song L., Chen Y., Ran H., Song J. (2017). Impact of ocean acidification on the early development and escape behavior of marine medaka (*Oryzias melastigma*). Mar. Environ. Res..

[10] Velez Z., Roggatz C.C., Benoit D.M., Hardege J.D., Hubbard P.C. (2019). Short- and medium-term exposure to ocean acidification reduces olfactory sensitivity in gilthead seabream. Front. Physiol..

[11] Lee Y.H., Jeong C.-B., Wang M., Hagiwara A., Lee J.-S. (2020). Transgenerational acclimation to changes in ocean acidification in marine invertebrates. Mar. Pollut. Bull..

[12] Parker L.M., Scanes E., O'Connor W.A., Ross P.M. (2021). Transgenerational plasticity responses of oysters to ocean acidification differ with habitat. J. Exp. Biol..

[13] Lee Y.H., Kim M.-S., Wang M., Bhandari R.K., Park H.G., Wu R.S.-S., Lee J.-S. (2022). Epigenetic plasticity enables copepods to cope with ocean acidification. Nat. Clim. Change.

[14] Vargas C.A., Lagos N.A., Lardies M.A., Duarte C., Manríquez P.H., Aguilera V.M., Broitman B., Widdicombe S., Dupont S. (2017). Species-specific responses to ocean acidification should account for local adaptation and adaptive plasticity. Nat. Ecol. Evol..

[15] Goncalves P., Jones D.B., Thompson E.L., Parker L.M., Ross P.M., Raftos D.A. (2017). Transcriptomic profiling of adaptive responses to ocean acidification. Mol. Ecol..

[16] Parker L.M., Ross P.M., O’Connor W.A. (2011). Populations of the Sydney rock oyster, *Saccostrea glomerata*, vary in response to ocean acidification. Mar. Biol..

[17] Tariel J., Plénet S., Luquet É. (2020). Transgenerational plasticity of inducible defences: Combined effects of grand-parental, parental and current environments. Ecol. Evol..

[18] Murray C.S., Malvezzi A., Gobler C.J., Baumann H. (2014). Offspring sensitivity to ocean acidification changes seasonally in a coastal marine fish. Mar. Ecol. Prog. Ser..

[19] Herman J.J., Sultan S.E. (2016). DNA methylation mediates genetic variation for adaptive transgenerational plasticity. Proc. R. Soc. B.

[20] Colicchio J.M., Herman J. (2020). Empirical patterns of environmental variation favor adaptive transgenerational plasticity. Ecol. Evol..

[21] Toni M., Angiulli E., Malavasi S., Alleva E., Cioni C. (2017). Variation in environmental parameters in research and aquaculture: effects on behaviour, physiology and cell biology of teleost fish. J. Aquacult. Mar. Biol..

[22] Whitfield A.K., Elliott M. (2002). Fishes as indicators of environmental and ecological changes within estuaries: a review of progress and some suggestions for the future. J. Fish. Biol..

[23] Vagner M., Zambonino-Infante J.-L., Mazurais D. (2019). Fish facing global change: are early stages the lifeline?. Mar. Environ. Res..

[24] Tseng Y.C., Hu M.Y., Stumpp M., Lin L.-Y., Melzner F., Hwang P.-P. (2013). CO_2_-driven seawater acidification differentially affects development and molecular plasticity along life history of fish (*Oryzias latipes*). Comp. Biochem. Physiol., Part A: Mol. Integr. Physiol..

[25] Hwang P.P. (2009). Ion uptake and acid secretion in zebrafish (*Danio rerio*). J. Exp. Biol..

[26] Thermes V., Lin C.C., Hwang P.P. (2010). Expression of *Ol-foxi3* and Na^+^/K^+^-ATPase in ionocytes during the development of euryhaline medaka (*Oryzias latipes*) embryos. Gene Expr. Patterns.

[27] Chang W.J., Hwang P.P. (2011). Development of zebrafish epidermis. Birth Defects Res., Part C.

[28] Heuer R.M., Grosell M. (2014). Physiological impacts of elevated carbon dioxide and ocean acidification on fish. Am. J. Physiol. Regul. Integr. Comp. Physiol..

[29] Esbaugh A.J. (2018). Physiological implications of ocean acidification for marine fish: emerging patterns and new insights. J. Comp. Physiol. B.

[30] Albright R., Langdon C. (2011). Ocean acidification impacts multiple early life history processes of the Caribbean coral *Porites astreoides*. Glob. Change Biol..

[31] Cohen-Rengifo M., Danion M., Gonzalez A.-A., Bégout M.-L., Cormier A., Noël C., Cabon J., Vitré T., Mark F.C., Mazurais D. (2022). The extensive transgenerational transcriptomic effects of ocean acidification on the olfactory epithelium of a marine fish are associated with a better viral resistance. BMC Genom..

[32] Sampaio E., Lopes A.R., Francisco S., Paula J.R., Pimentel M., Maulvault A.L., Repolho T., Grilo T.F., Pousão-Ferreira P., Marques A., Rosa R. (2018). Ocean acidification dampens physiological stress response to warming and contamination in a commercially-important fish (*Argyrosomus regius*). Sci. Total Environ..

[33] Furukawa F., Tseng Y.-C., Liu S.-T., Chou Y.-L., Lin C.-C., Sung P.-H., Uchida K., Lin L.-Y., Hwang P.-P. (2015). Induction of phosphoenolpyruvate carboxykinase (PEPCK) during acute acidosis and its role in acid secretion by V-ATPase-expressing ionocytes. Int. J. Biol. Sci..

[34] Hwang P., Perry S. (2010). Fish Physiology.

[35] Trueman C., Johnston G., O'hea B., MacKenzie K. (2014). Trophic interactions of fish communities at midwater depths enhance long-term carbon storage and benthic production on continental slopes. Proc. R. Soc. B.

[36] Tidwell J.H., Allan G.L. (2001). Fish as food: aquaculture's contribution. Ecological and economic impacts and contributions of fish farming and capture fisheries. EMBO Rep..

[37] Mu J., Jin F., Wang J., Zheng N., Cong Y. (2015). Effects of CO_2_-driven ocean acidification on early life stages of marine medaka (*Oryzias melastigma*). Biogeosciences.

[38] Liu S.-T., Horng J.-L., Lin L.-Y. (2022). Role of the basolateral Na^+^/H^+^ exchanger-2 (NHE2) in ionocytes of seawater-acclimated medaka (*Oryzias latipes*). Front. Physiol..

[39] Liu S.-T., Horng J.-L., Chen P.-Y., Hwang P.-P., Lin L.-Y. (2016). Salt secretion is linked to acid-base regulation of ionocytes in seawater-acclimated medaka: new insights into the salt-secreting mechanism. Sci. Rep..

[40] Tresguerres M., Hamilton T.J. (2017). Acid–base physiology, neurobiology and behaviour in relation to CO_2_-induced ocean acidification. J. Exp. Biol..

[41] McGaughey D.M., Abaan H.O., Miller R.M., Kropp P.A., Brody L.C. (2014). Genomics of CpG methylation in developing and developed zebrafish. G3.

[42] Li S., He F., Wen H., Li J., Si Y., Liu M., Huang Y., Meng L. (2017). Low salinity affects cellularity, DNA methylation, and mRNA expression of igf1 in the liver of half smooth tongue sole (*Cynoglossus semilaevis*). Fish Physiol. Biochem..

[43] Andersson A., Kline D., Edmunds P., Archer S., Bednaršek N., Carpenter R., Chadsey M., Goldstein P., Grottoli A., Hurst T. (2015). Understanding ocean acidification impacts on organismal to ecological scales. Oceanography.

[44] De Wit P., Dupont S., Thor P. (2016). Selection on oxidative phosphorylation and ribosomal structure as a multigenerational response to ocean acidification in the common copepod *Pseudocalanus acuspes*. Evol. Appl..

[45] Lohbeck K.T., Riebesell U., Reusch T.B.H. (2014). Gene expression changes in the coccolithophore Emiliania huxleyi after 500 generations of selection to ocean acidification. Proc. R. Soc. B.

[46] Michael K., Kreiss C.M., Hu M.Y., Koschnick N., Bickmeyer U., Dupont S., Pörtner H.O., Lucassen M. (2016). Adjustments of molecular key components of branchial ion and pH regulation in Atlantic cod (*Gadus morhua*) in response to ocean acidification and warming. Comp. Biochem. Physiol. B Biochem. Mol. Biol..

[47] Malte H., Lykkeboe G., Wang T. (2021). The magnitude of the Bohr effect profoundly influences the shape and position of the blood oxygen equilibrium curve. Comp. Biochem. Physiol. Mol. Integr. Physiol..

[48] Jensen F.B. (2004). Red blood cell pH, the Bohr effect, and other oxygenation-linked phenomena in blood O_2_ and CO_2_ transport. Acta Physiol. Scand..

[49] Liang P., Saqib H.S.A., Ni X., Shen Y. (2020). Long-read sequencing and *de novo* genome assembly of marine medaka (*Oryzias melastigma*). BMC Genom..

[50] Kim H.S., Lee B.Y., Han J., Jeong C.B., Hwang D.S., Lee M.C., Kang H.M., Kim D.H., Lee D., Kim J. (2018). The genome of the marine medaka *Oryzias melastigma*. Mol. Ecol. Resour..

[51] Olsen R.W., Sieghart W. (2009). GABAA receptors: subtypes provide diversity of function and pharmacology. Neuropharmacology.

[52] Kaila K., Ruusuvuori E., Seja P., Voipio J., Puskarjov M. (2014). GABA actions and ionic plasticity in epilepsy. Curr. Opin. Neurobiol..

[53] Forsgren E., Dupont S., Jutfelt F., Amundsen T. (2013). Elevated CO_2_ affects embryonic development and larval phototaxis in a temperate marine fish. Ecol. Evol..

[54] Nagelkerken I., Munday P.L. (2016). Animal behaviour shapes the ecological effects of ocean acidification and warming: moving from individual to community-level responses. Glob. Change Biol..

[55] Clark T.D., Raby G.D., Roche D.G., Binning S.A., Speers-Roesch B., Jutfelt F., Sundin J. (2020). Ocean acidification does not impair the behaviour of coral reef fishes. Nature.

[56] Clark T.D., Raby G.D., Roche D.G., Binning S.A., Speers-Roesch B., Jutfelt F., Sundin J. (2020). Reply to: Methods matter in repeating ocean acidification studies. Nature.

[57] Lim Y.-K., Cheung K., Dang X., Roberts S.B., Wang X., Thiyagarajan V. (2021). DNA methylation changes in response to ocean acidification at the time of larval metamorphosis in the edible oyster, *Crassostrea hongkongensis*. Mar. Environ. Res..

[58] Liew Y.J., Howells E.J., Wang X., Michell C.T., Burt J.A., Idaghdour Y., Aranda M. (2020). Intergenerational epigenetic inheritance in reef-building corals. Nat. Clim. Change.

[59] Jablonka E. (2017). The evolutionary implications of epigenetic inheritance. Interface focus.

[60] Stajic D., Perfeito L., Jansen L.E.T. (2019). Epigenetic gene silencing alters the mechanisms and rate of evolutionary adaptation. Nat. Ecol. Evol..

[61] Lynch M. (2007). The frailty of adaptive hypotheses for the origins of organismal complexity. Proc. Natl. Acad. Sci. USA.

[62] Cropley J.E., Dang T.H.Y., Martin D.I.K., Suter C.M. (2012). The penetrance of an epigenetic trait in mice is progressively yet reversibly increased by selection and environment. Proc. R. Soc. B.

[63] Wolf C., Linden D.E.J. (2012). Biological pathways to adaptability–interactions between genome, epigenome, nervous system and environment for adaptive behavior. Genes Brain Behav..

[64] Donohoe D.R., Bultman S.J. (2012). Metaboloepigenetics: interrelationships between energy metabolism and epigenetic control of gene expression. J. Cell. Physiol..

[65] Furusawa C., Kaneko K. (2013). Epigenetic feedback regulation accelerates adaptation and evolution. PLoS One.

[66] Ghedini G., Connell S.D. (2017). Moving ocean acidification research beyond a simple science: investigating ecological change and their stabilizers. Food Webs.

[67] Riebesell U., Bach L.T., Bellerby R.G.J., Monsalve J.R.B., Boxhammer T., Czerny J., Larsen A., Ludwig A., Schulz K.G. (2017). Competitive fitness of a predominant pelagic calcifier impaired by ocean acidification. Nat. Geosci..

[68] Teske P.R., Zardi G.I., McQuaid C.D., Nicastro K.R. (2013). Two sides of the same coin: extinctions and originations across the Atlantic/Indian Ocean boundary as consequences of the same climate oscillation. Front. Biogeogr..

[69] Huang S., Roy K., Valentine J.W., Jablonski D. (2015). Convergence, divergence, and parallelism in marine biodiversity trends: Integrating present-day and fossil data. Proc. Natl. Acad. Sci. USA.

[70] Lewis E., Wallace D. (1998).

[71] Sarazin G., Michard G., Prevot F. (1999). A rapid and accurate spectroscopic method for alkalinity measurements in sea water samples. Water Res..

[72] Holmes R.M., Aminot A., Kérouel R., Hooker B.A., Peterson B.J. (1999). A simple and precise method for measuring ammonium in marine and freshwater ecosystems. Can. J. Fish Aquat. Sci..

[73] Ge S.X., Son E.W., Yao R. (2018). iDEP: an integrated web application for differential expression and pathway analysis of RNA-Seq data. BMC Bioinf..

[74] Zhang Z., Hu J. (2007). Development and validation of endogenous reference genes for expression profiling of medaka (*Oryzias latipes*) exposed to endocrine disrupting chemicals by quantitative real-time RT-PCR. Toxicol. Sci..

[75] Fox J., Weisberg S. (2019). https://CRAN.R-project.org/package=car.

[76] R Core Team (2024). R: A language and environment for statistical computing.

[77] Kassambara A. (2023). rstatix: Pipe-friendly framework for basic statistical tests. https://cran.r-project.org/package=rstatix.

