# Supplementary material for: Epigenetic insights into physiological resilience: Multigenerational readouts of CO2-induced seawater acidification effects on fish embryos
Source: iScience. 2025 Jul 26;28(9):113187. doi: 10.1016/j.isci.2025.113187 (PMC12362705; doi:10.1016/j.isci.2025.113187)
Supplement: Document S1. Figures S1–S4 [file mmc1.pdf]

iScience, Volume *28*

Supplemental information

Epigenetic insights into physiological resilience: Multigenerational readouts of CO_2-induced seawater acidification effects on fish embryos

Tzu-Yen Liu, Jia-Jiun Yan, Ying-Jey Guh, Oki Hayasaka, Li-Yih Lin, Pung-Pung Hwang, Guan-Chung Wu, Ming-Tsung Chung, and Yung-Che Tseng

Supplemental Figure S1

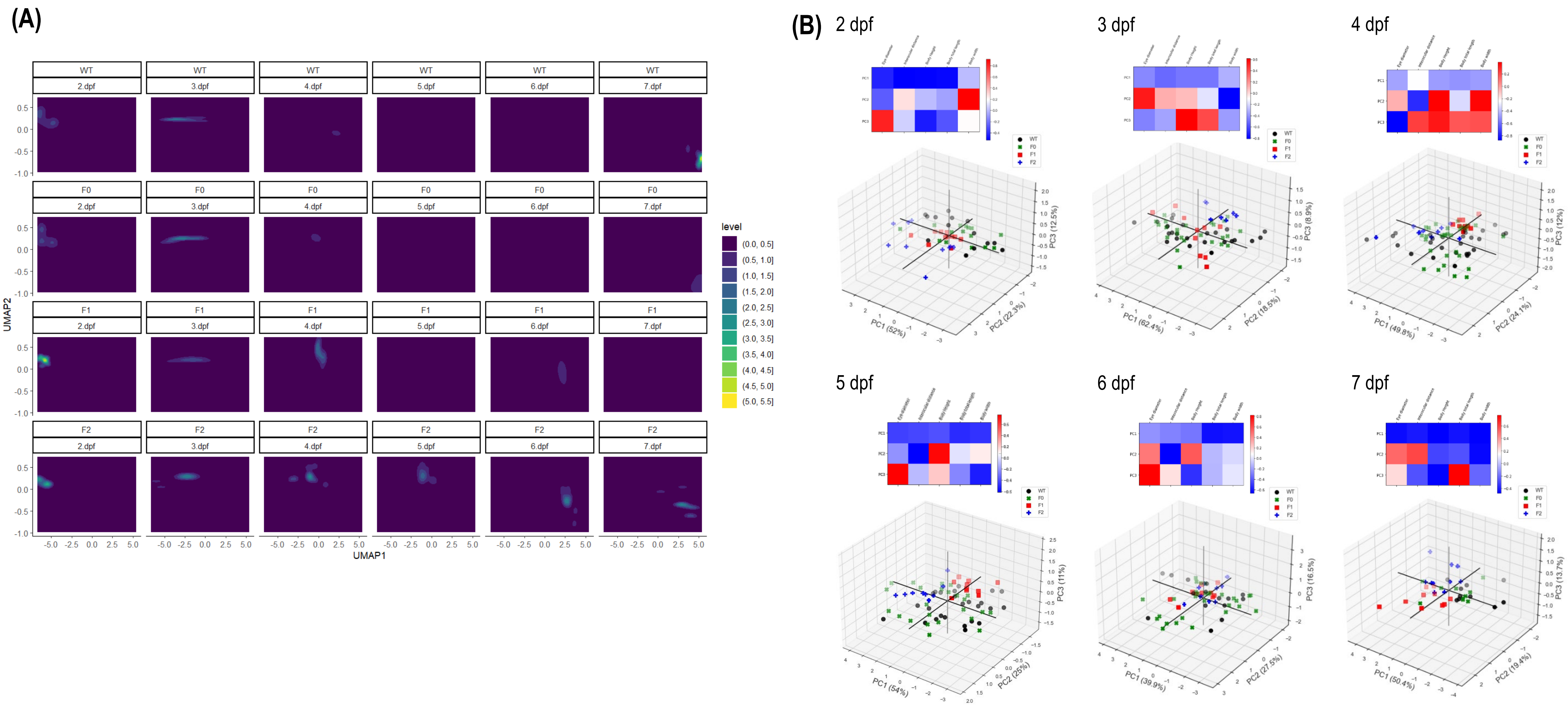

Supplemental Figure S1: Developmental trajectory analysis of marine medaka embryos from 2 to 7 days post-fertilization (dpf) under control and elevated CO_2–induced acidified conditions.

(A) Uniform Manifold Approximation and Projection (UMAP) analysis of growth parameters, including interocular distance, eye diameter, body total length, body height, and body width, across multiple generations (WT, F0, F1, F2) and developmental stages (2-7 dpf). The color gradient represents the Kernel Density Estimation (KDE) of data points, indicating the distribution of growth patterns within each group. The UMAP analysis reveals increasing divergence in growth trajectories among groups. (B) Principal Component Analysis (PCA) of growth parameters at each developmental stage, dissecting the contributions of principal components (PC1, PC2, and PC3). The CO_2-acclimated F0 generation exhibits phenotypic similarities to the wild type (WT), while the F1 and F2 generations under prolonged CO_2 exposure demonstrate distinct separations from both WT and F0 groups, indicating the cumulative impact of acidified conditions on developmental trajectories. These analyses provide a comprehensive assessment of the dynamic growth changes in marine medaka larvae under varying CO_2 conditions, highlighting the transgenerational effects of environmental stress on early life stages.

Supplemental Figure S2

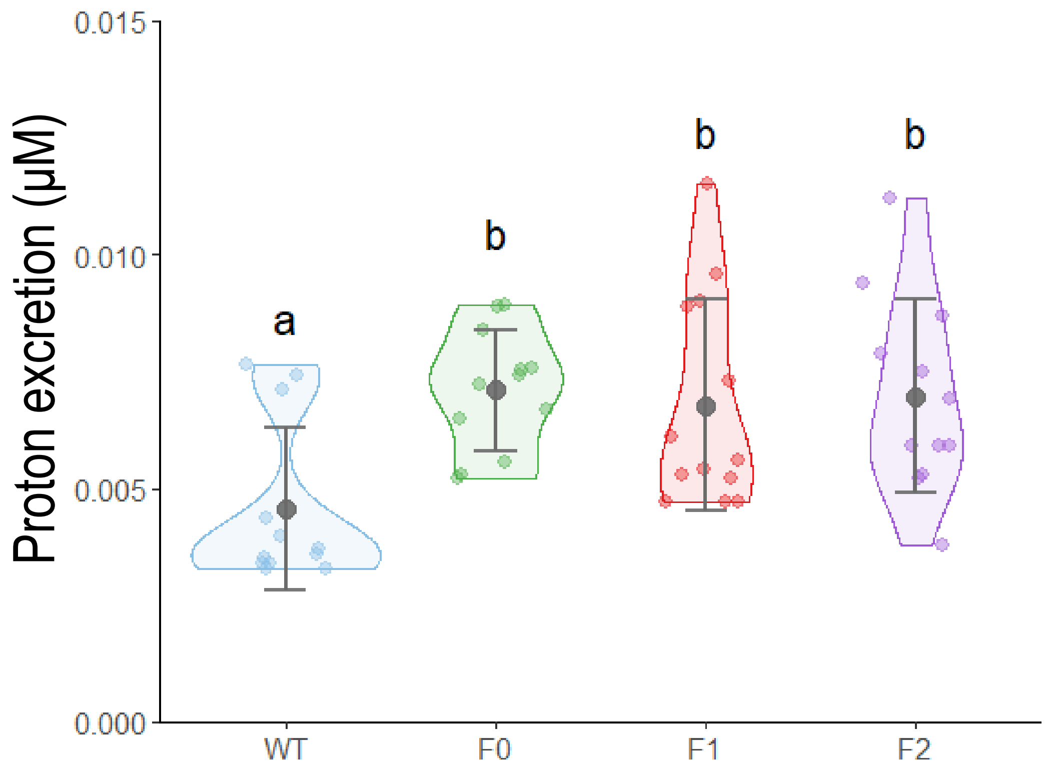

Supplemental Figure S2: Proton excretion capacity from yolk sac epithelium of 5 dpf marine medaka embryos under control and elevated CO_2–induced acidified conditions.

Proton excretion capacity of 5 dpf embryos in control and acidified groups. Data are expressed as mean ± SD. Different letters indicate significant differences among different treatment groups (one-way ANOVA, Tukey's pairwise comparisons).

Supplemental Figure S3

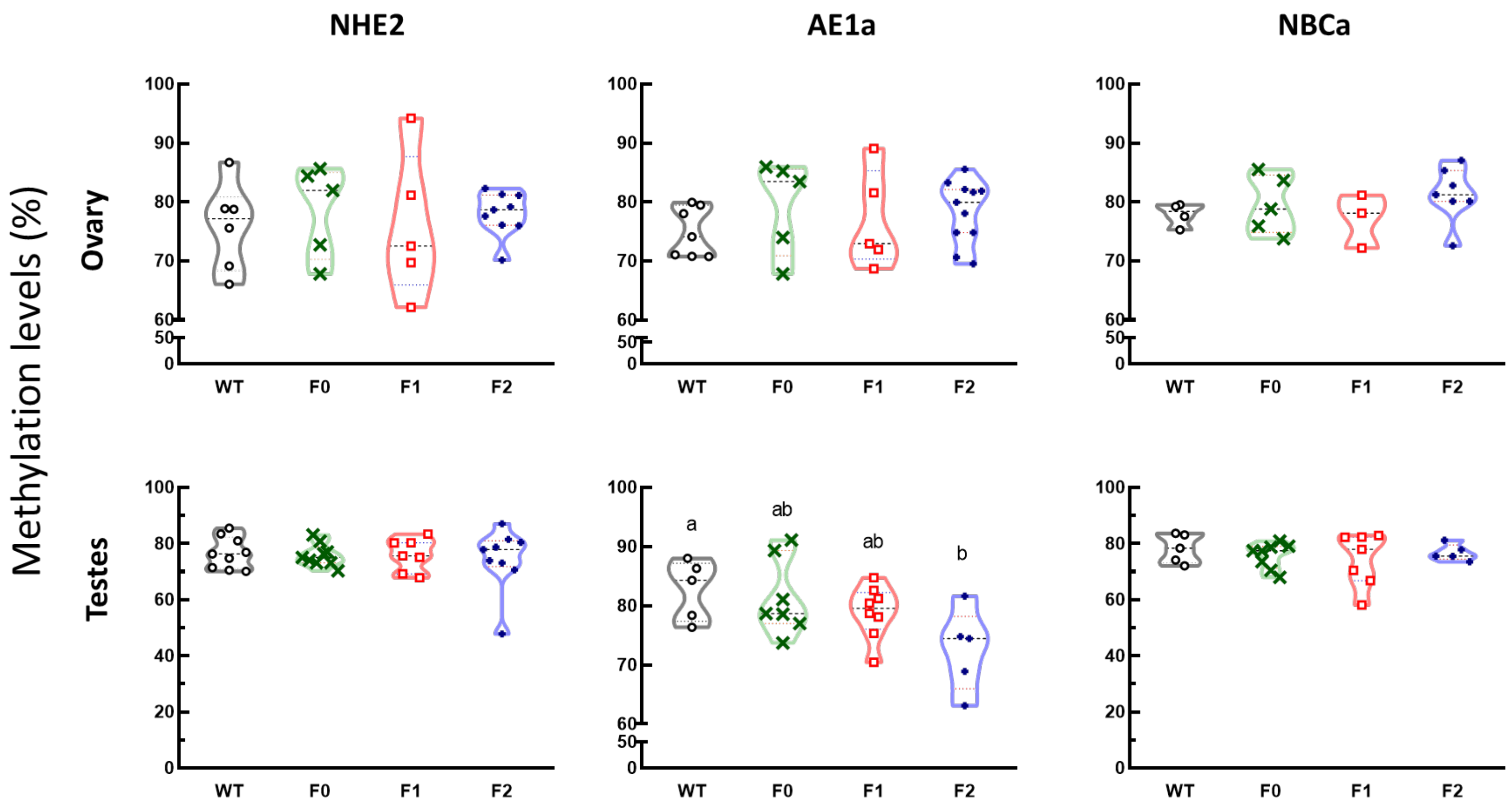

Supplemental Figure S3 DNA methylation patterns at the promoter regions of selected acid-base regulation genes (NHE2, AE1a, and NBCa) in the gonads of adult marine medaka across multiple generations exposed to control and elevated CO_2–induced acidified conditions. Methylation levels were quantified using Methylated DNA Immunoprecipitation (MeDIP) and analyzed by one-way ANOVA followed by Tukey's post hoc test to assess differences among groups (WT, F0, F1, and F2). The ovaries exhibited no significant variation in methylation patterns across all conditions, indicating a lack of epigenetic response in female gonads. In contrast, the testes displayed a significant difference in AE1a methylation levels between the wildtype (WT) and the F2 generation, suggesting generation-specific epigenetic modifications linked to prolonged exposure to acidified environments. These results underscore the differential epigenetic responses in male and female gonads to multigenerational exposure to environmental stressors, providing insights into the potential role of epigenetic mechanisms in shaping the adaptive capacity of marine organisms to changing ocean conditions.

Supplemental Figure S4

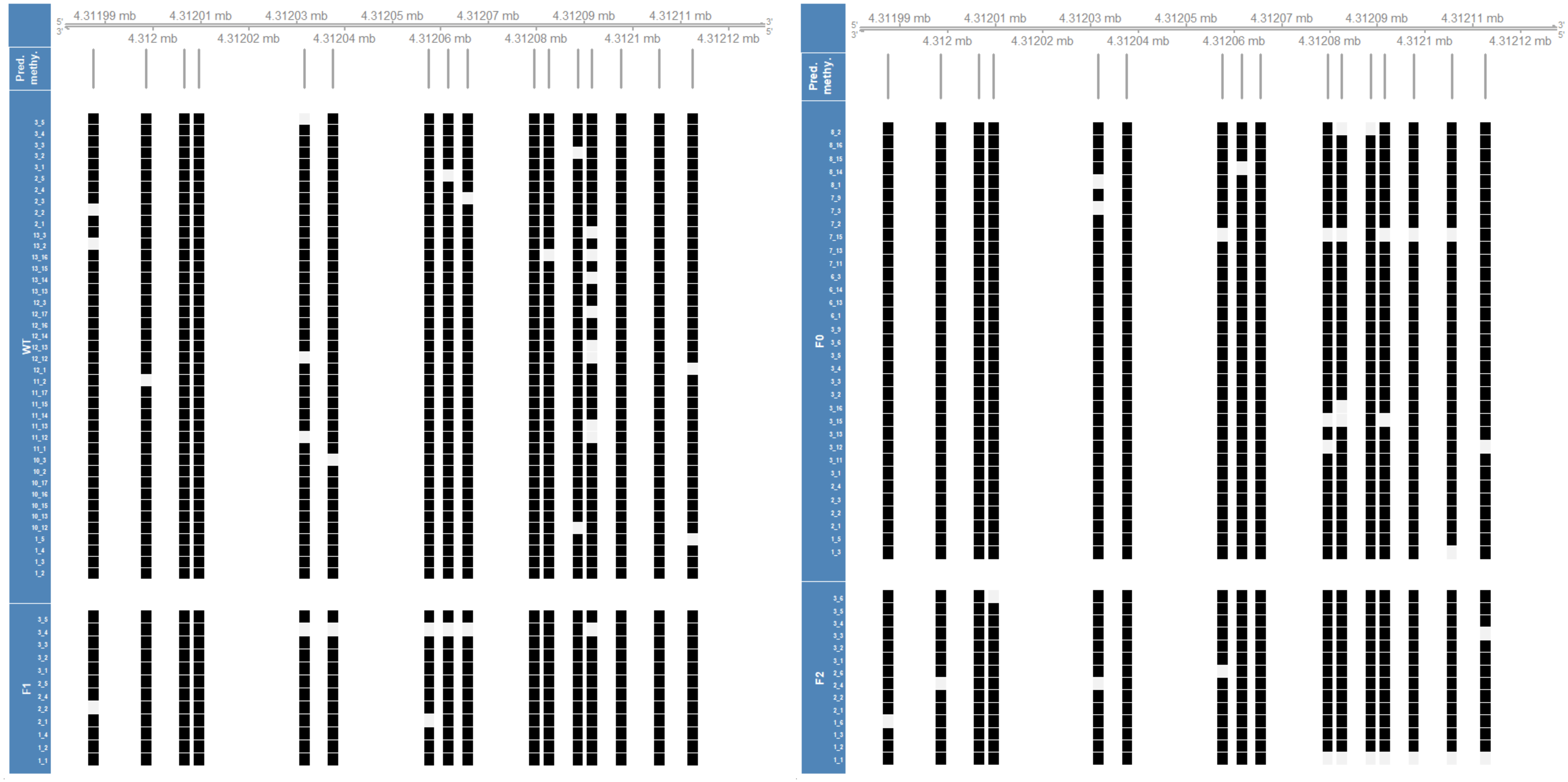

Supplemental Figure S4 High-resolution analysis of the methylation landscape at the promoter region of the AE1a gene in marine medaka embryos across multiple generations exposed to control and elevated CO_2–induced acidified conditions.

The figure presents the methylation dynamics captured through bisulfite sequencing, with each row representing an individual sequencing read and each column corresponding to a specific CpG site within the promoter region. Methylated cytosines are depicted as black squares, while unmethylated cytosines are represented by white squares. The spatial distribution of methylated versus unmethylated sites provides a detailed view of the epigenetic regulation of AE1a under various experimental conditions. The stark contrast between black and white squares effectively illustrates the presence or absence of methylation at specific sites, facilitating a clear understanding of the gene's epigenetic landscape. This high-resolution analysis complements the quantitative assessment of methylation levels (Fig. 3C) and offers a visual representation of the dynamic epigenetic changes associated with transgenerational exposure to acidified environments, highlighting the potential role of epigenetic mechanisms in shaping the adaptive responses of marine organisms to global change.
